# Magnetic Sensors Based on Amorphous Ferromagnetic Materials: A Review

**DOI:** 10.3390/s151128340

**Published:** 2015-11-11

**Authors:** Carlos Morón, Carolina Cabrera, Alberto Morón, Alfonso García, Mercedes González

**Affiliations:** Sensors and Actuators Group, Department of Building Technology, Polytechnic University of Madrid, 28040 Madrid, Spain; E-Mails: carolina.cmertens@alumnos.upm.es (C.C.); 100292194@alumnos.uc3m.es (A.M.); alfonso.garciag@upm.es (A.G.); mer.gonzalez@upm.es (M.G.)

**Keywords:** magnetic sensor, amorphous ribbons, amorphous wire, ferromagnetic material, magnetic core, magnetometer, magnetoimpedance, bistability, security system, weapon detectors

## Abstract

Currently there are many types of sensors that are used in lots of applications. Among these, magnetic sensors are a good alternative for the detection and measurement of different phenomena because they are a “simple” and readily available technology. For the construction of such devices there are many magnetic materials available, although amorphous ferromagnetic materials are the most suitable. The existence in the market of these materials allows the production of different kinds of sensors, without requiring expensive manufacture investments for the magnetic cores. Furthermore, these are not fragile materials that require special care, favouring the construction of solid and reliable devices. Another important feature is that these sensors can be developed without electric contact between the measuring device and the sensor, making them especially fit for use in harsh environments. In this review we will look at the main types of developed magnetic sensors. This work presents the state of the art of magnetic sensors based on amorphous ferromagnetic materials used in modern technology: security devices, weapon detection, magnetic maps, car industry, credit cards, *etc.*

## 1. Introduction

For a robot to perform tasks such as location and dimensioning of objects in a work place, the action of a sensor is needed. The data collected by the sensor also feed back to the environment, enabling the robot to examine them and take decisions.

The use of external sensor mechanisms allows a robot to interact with its environment in a flexible way, in contrast to preprogrammed actions in which a robot is taught to carry out repetitive tasks out of a set of scheduled functions. Although current industrial robots operate most frequently according to the latter mechanism, the use of sensor technologies to equip machines with a higher intelligence level when dealing with their environment is actually a topic of active research and development in the field of robotics [1,2,3].

Among the current sensors that can be handled, magnetic sensors are a good alternative for the detection and measurement of various phenomena because of their “simple” technology and because they can be easily acquired. A wide range of magnetic materials is available to build these types of devices, among which amorphous ferromagnetic materials should be highlighted [4,5,6]. Since these materials are available on the market, the production of different kinds of sensors can be realized without the expensive investments needed for the manufacture of their magnetic cores. These sensors are not fragile and do not require special care, which enables the construction of very solid and reliable devices. Another important feature in their behalf is that these sensors can be developed without electric contact between the measuring device and the sensor, making them especially fit for use in harsh environments. Magnetic sensors work basically by detecting [7,8]:

(a) Variations of magnetic core permeability produced by the parameter to be measured.
(b) Changes in some physical parameters produced by changes of the magnetization direction.
(c) Mutual induction changes between two circuits produced by geometric modifications of the magnetic core positioning.

The permeability of magnetic materials depends greatly on their magnetic anisotropy, on the difference between the direction of the applied field and the anisotropy direction of the material, as well as on their homogeneity, magnetization state, frequency of the applied field, surface roughness and form. In the development of a sensor for measuring a physical parameter, it is necessary that changes of magnetic permeability or of the magnetization direction caused by a change in the parameter to be measured are as large as possible. Amorphous and nanocrystalline materials meet these requirements particularly well.

## 2. Sensors Based on Magnetostrictive Effects

Currently there are a lot of sensors that use the magnetoelastic effects of magnetic materials. Their construction is based upon several properties such as:

- Variation of magnetic material susceptibility when a mechanical stress is applied.
- Length variation of magnetic materials when the magnetization direction changes.
- Modification of the Young’s modulus of magnetic materials by varying the magnetization state.

### 2.1. Stress Sensor

#### 2.1.1. Theory

The application of a mechanical stress, σ, to a ferromagnetic material induces an anisotropy whose energy density is K = (3/2) λ_s_ σ in perpendicular or parallel direction to this stress depending on the positive or negative sign of the magnetostriction constant of the material, λ_s_. Therefore, a mechanical stress will cause a change in the susceptibility of the material that can be used to measure stress, strain, torques, forces, *etc.* [9].

The susceptibility χ of a ferromagnetic material of λ_s_ > 0 with an initial anisotropy K perpendicular to the applied stress, which is magnetized with a magnetic field applied along to the stress direction, can be evaluated by:

(1) $$\chi \;=\;\frac{\mu_{0}M_{S}^{2}}{\left(2\;K-3\;\lambda_{S}\;\sigma \right)}\;$$

**Figure 1:**
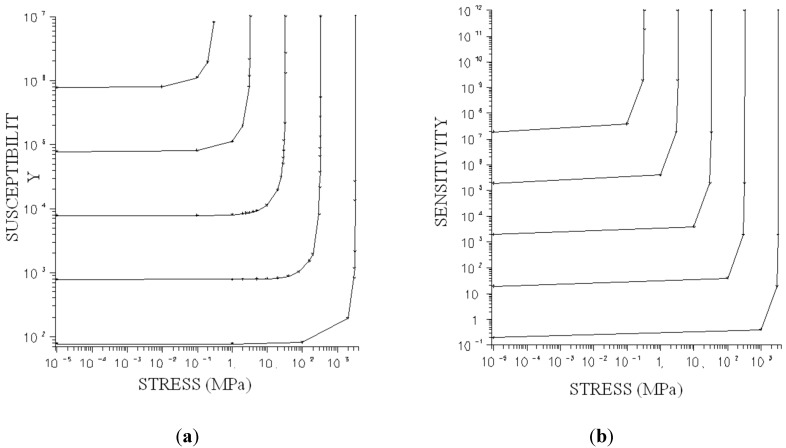
Susceptibility (**a**) and sensitivity (**b**) variation with applied stress.

Figure 1a shows the susceptibility (χ) depending on the stress applied (σ). When the resulting anisotropy takes the direction of the applied field, the material reaches its maximum permeability due to a process of magnetic walls displacement. This maximum permeability limits the maximum sensitivity of the device and is dependent on the above parameters. The maximum sensitivity of the device is obtained for extremely small densities (K), as is shown in Figure 1b, where the sensitivity curve as a function of the stress applied is represented.

In this form the sensitivity is:

(2) $$\frac{d\chi }{d\sigma }=\;\left[\frac{\mu_{0}M_{S}^{2}}{{\left(2\;K-3\;\lambda_{S}\;\sigma \right)}^{2}}\right]\;3\;\lambda_{S}$$

**Figure 2:**
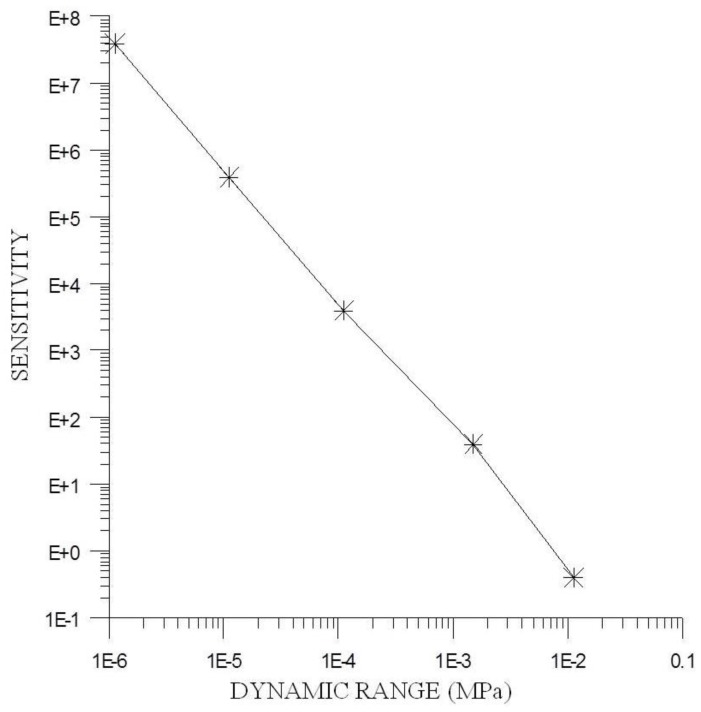
Sensitivity *vs.* dynamic range.

In amorphous materials K is well controlled. Accordingly, it is possible to obtain devices with sensitivities that are equivalent to the best semiconductor strain gauge, although in this form the dynamic range of the devices decreases (Figure 2). Therefore it is advisable to select materials with high λ_s_ to achieve acceptable dynamic ranges without decreasing their sensitivity.

#### 2.1.2. Applications

(a) Torque Sensors

**Figure 3:**
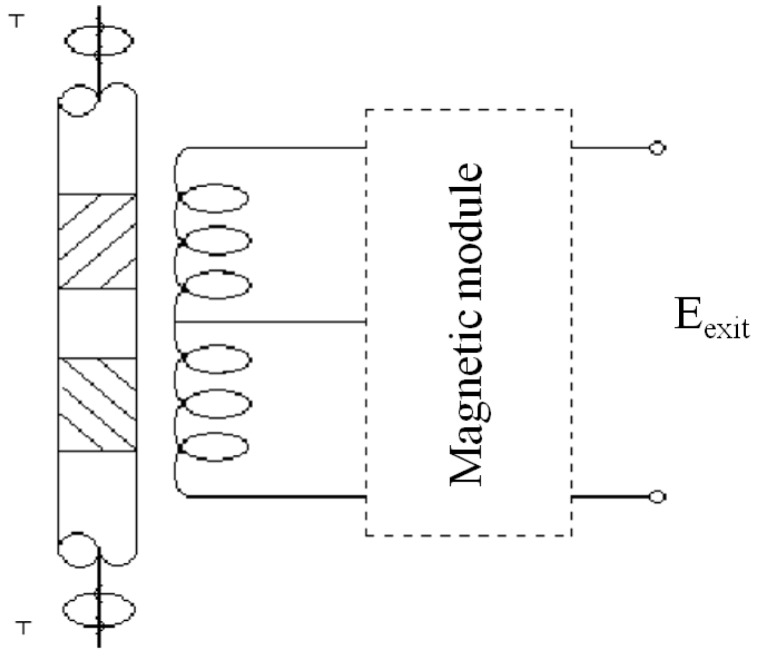
Torque sensor.

Figure 3 shows a typical application of these devices for the measurement of torques on rotary axes without electrical contacts [10,11,12]. For sensors with the shape of layers or thin tapes, magnetic materials are used in a similar way as extensometric bands are: two sets of equal samples, perpendicularly placed between themselves in an angle of 45° with the rotation axis, are stuck to the shaft in which the torque is to be measured. If the shaft undergoes torsion, one set of samples is tensed whereas the other set is compressed, modifying in a different way the permeability of both of them and inducing an electro-motive force (e.m.f.) on the series connected secondary reels. If there is no torsion both have the same permeability being the outgoing signal zero. If, instead, there is torsion a signal proportional to the relative permeability change is recorded. This system allows one to measure torques on rotary axes without using sliding contacts, which always give problems.

The main drawback of this measuring system are the induced stresses on the samples when gluing the layers to the base; this effect can be avoided by using ceramic glues and annealing afterwards the system in order to remove the induced stresses.

(b) Muscular Activity Measurement Sensor

This sensor has been developed by Pina *et al.* [13] to detect and measure the muscle activity in the throat, in order to remedy partial vocal cord paralysis. This ailment concerns the paralysis of one of the vocal cords due to its partial or total weakness.

The voice is produced by simultaneous vibration of both vocal cords. An air column coming from the lungs passes through the vocal cords at a certain pressure, causing a natural vibration. In order to reach the right pressure level, the vocal cords move towards each other until reaching the so-called midline, closing the air flow properly. This is the normal function of a healthy throat.

In case of paralysis, the damaged cord stays far away from the midline being unable to move itself to reach the right pressure level. As a result, the cords do not vibrate and the phonation is not totally possible.

The manufactured device consists of two clearly different parts. The first element is a magnetostrictive sensor which is inserted inside the healthy vocal cord. This sensor can detect the deformation and tension inside de muscle during the normal activity of the vocal cord. The sensor will + respond to a certain deformation with an electric signal which will start up the device's second element. The second element consists of an actuator composed of a set of electrodes connected to the vocal cord nerve. The electrical stimulation of the unhealthy vocal cord will be controlled by the responses off the magnetoelastic sensor located on the cord. This way both vocal cords will move simultaneously and symmetrically. The system is regulated by a feedback control that includes a second magnetoelastic sensor inserted inside de damaged vocal cord.

The sensor manufactured for this application was designed to respond to stresses applied in any direction of the insertion plane. Thus the sensor core is composed of a magnetostrictive tape in the shape of a ring with two rolled coils around the core: a drive coil of sine waved voltage within a range of hundreds of kHz frequencies and a secondary coil. The sensor is covered with biocompatible silicone and the sensors head has a 3 mm diameter. The optimal work frequency obtained is of 500 kHz.

The sensor has been tried on dogs. The experiment shows clearly two signal peaks that correspond respectively to deglutition and phonation.

(c) Deformation Sensor

**Figure 4:**
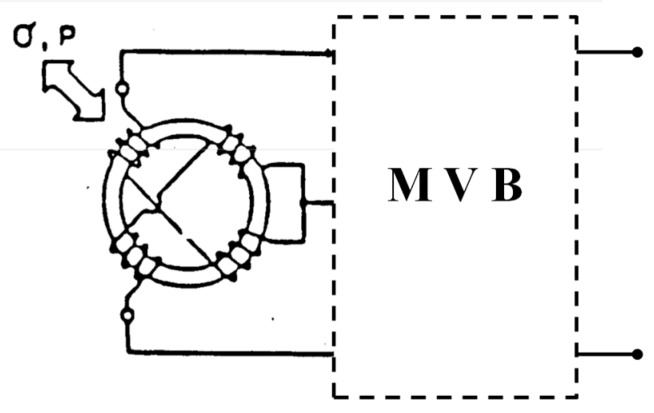
Sensor used as anti-aircraft position system with a multivibrator bridge (MVB).

Another possible application of this effect is shown in Figure 4. This device [14,15,16] uses a several layers round core with multiple windings. Any pressure being exerted on the torus modifies the self-induction in the windings, which allows a very accurate measuring of deformations. The sensitivity of the sensor is of 350 mV/V within a range of 10 mg–30 g, showing stability at a temperature of 0.02%FS/°C (FS is full scale) and a maximum operating temperature of 190 °C.

(d) Current Sensor

There are also sensors to measure magnetic fields, current capacities, deformations, *etc.* that have been developed using the inverse effect as earlier described: the change in length of ferromagnetic materials when varying their magnetic direction. Optic fibres with nickel cover, acted upon by a magnetic field, vary in length when the length of their cover is altered Through interferometric measures, it is possible to determine with great accuracy the variation of that length which is proportional to the applied magnetic field [17,18].

**Figure 5:**
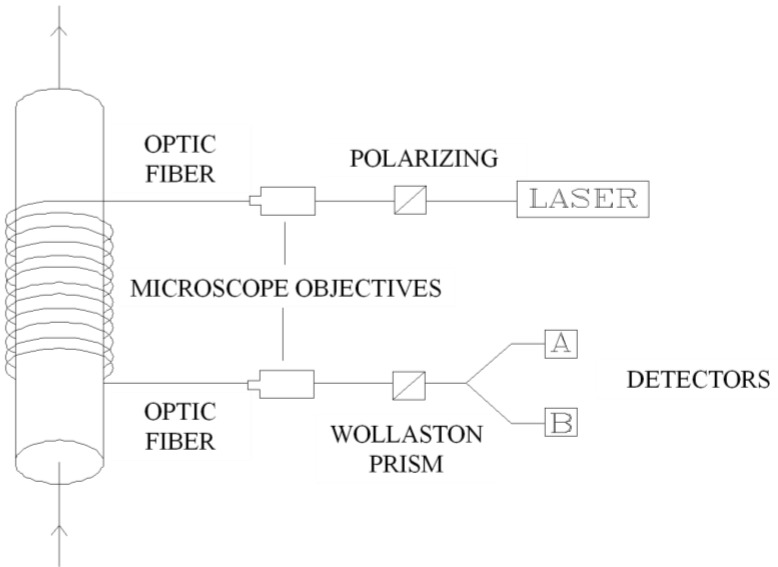
Current sensor based on material length variation with magnetic direction.

Figure 5 shows a current sensor based on this effect. With some slight changes it can also be used to measure magnetic fields. Position sensors are measured with an optic fibre sensor through a field created by a permanent magnet stuck to the object of which the position is to be determined.

(e) Magnetic Field Sensor

**Figure 6:**
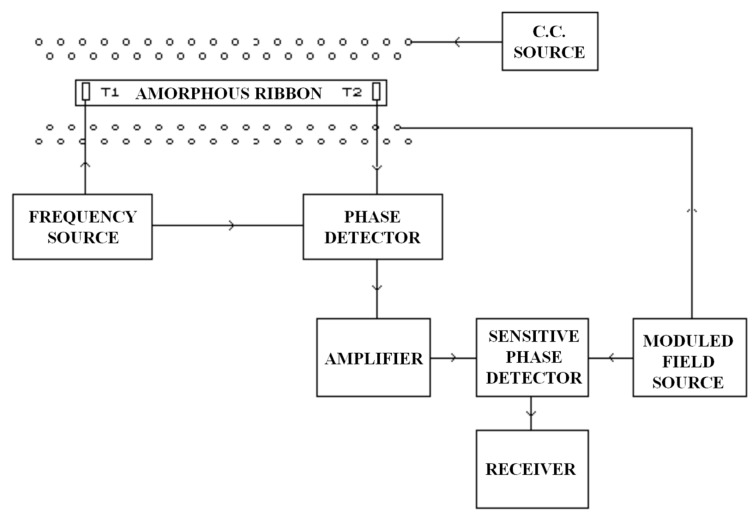
Sensor based on a Young modulus change.

Finally, magnetometers with a much lower sensitivity have been developed. They are based on speed variations of acoustic waves that are conveyed through a tape of amorphous material when their magnetization state and, consequently, their Young modulus changes by the action of an outside field [19]. The detection of this speed variation is done measuring the phase changing of the elastic waves emitted by the source and those collected by a receiver (Figure 6).

The wave phase change will be according to:

(3) $$\Delta \text{Φ}\;=\;\pi \;\left(\frac{D\;\omega \;K}{c}\right)\;\Delta H$$

where K is a parameter in proportion to the Young modulus, D the distance between transducers and C the acoustic wave speed. This sensor has an estimated sensitivity of 1 pT (ΔY = 0.05, D = 1 m, ω = 20 MHz, c = 3 Km/s) and an experimental sensitivity of 2.5 nT.

## 3. Sensors Based on the Nonlinearity of Magnetization Processes

### 3.1. Saturated Core Magnetometers

#### 3.1.1. Theory

**Figure 7:**
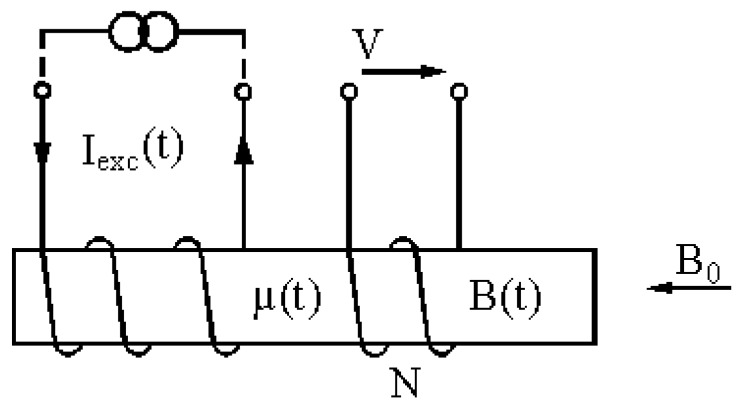
Magnetometer sensor with single core [20].

A saturated core sensor (or fluxgate magnetometer) is a device which uses the periodic saturation of its ferromagnetic core (or cores) to produce an output signal with harmonic components that are nearly proportional to the device’s external magnetic fields. The ferromagnetic core is periodically saturated through a current applied to an exciting winding, while the net variation of the core flow is observed with the help of a measuring winding. If there is no external magnetic field, the electromotive force induced into the measuring winding contains at least the odd harmonics of the exciting current, unless the device is configured (as is usual) in a way that keeps the mutual inductance between exciting and measuring winding to a minimum, in which case the odd harmonics also near zero. When an external magnetic field is applied, the operation becomes asymmetrical, producing even harmonics in the measuring winding [20]. Consequently, this device uses the (nonlinear) magnetic features of the ferromagnetic core material to form a directional sensor which measures the component of an external field parallel to the measuring coil’s (Figure 7) axes.

#### 3.1.2. Double Core Fluxgate

**Figure 8:**
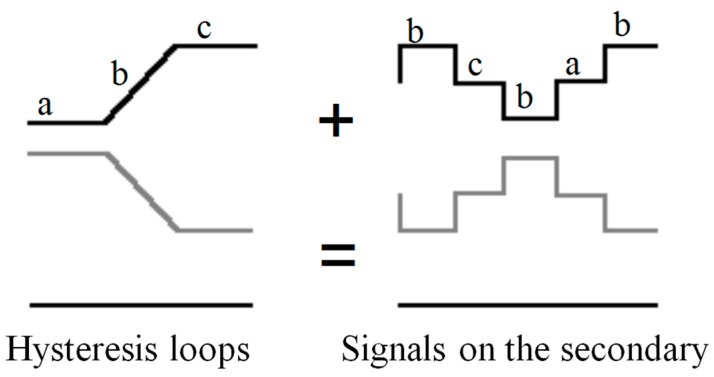
Signal of each core without external field applied.

This type of sensor consists of two primary coils connected in opposite series with two identical cores inside, both of them surrounded by the secondary coil. If through the coil flows a sinusoidal current, the cores will magnetize in opposite direction (because they are coiled in series-opposition). Figure 8 shows the signals that each core introduces in the secondary coil, in the absence of an external (black and grey) field, supposing that the primary flow is sufficient to saturate the core at some time. The total signal beneath will be seen in the secundary coil, which is the sum of both. Indeed, if the cores are identical the total e.m.f. induced equals zero.

**Figure 9:**
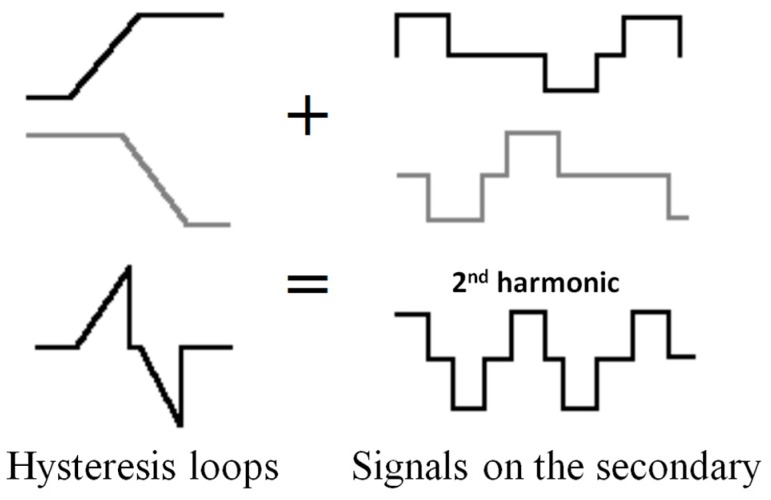
Signal of each core applying an external field.

The situation is completely different when an external field is applied. In this event, one core will remain more time saturated than the other one during a half-cycle, while during the next half-cycle the opposite will happen. In this way, the sum of the two signals in the secondary coil is not voided, giving a signal with components whose frequency doubles the frequency of the exciting current (see Figure 9).

The amplitude of this second harmonic induced in the secondary coil will be directly proportional to the amplitude of the external magnetic field (provided that one of the cores is not permanently saturated: no linear range). The signals and cycles shown in this paper will not much look like those of the drawings, as real hysteresis loops can never be so linear.

If toroids are used instead of longitudinal cores, it is possible to manufacture sensors to measure magnetic fields in orthogonal directions [21,22]. With an accurate set of three magnetometers, the three components of any magnetic field can be easily measured.

**Figure 10:**
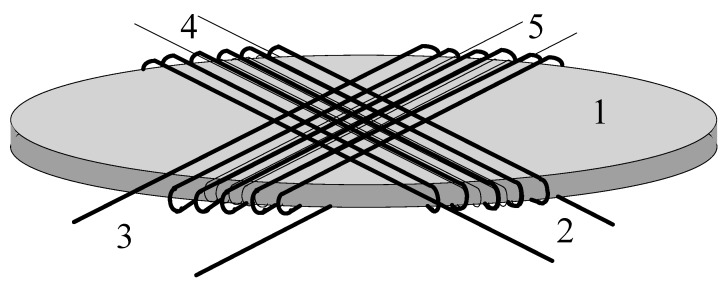
Schematic view of two-axis sensor unit. 1: Amorphous disc. 2/3: Orthogonal primary coils. 4/5: Orthogonal pick-up coils [21].

The linearity of this device can be greatly improved by adding a fourth reel (Figure 10) which creates a magnetic field that compensates the field to be detected, this means: use the sensor as a zero machine. Without a compensating reel these devices can measure fields up to 100 nT whereas with a compensating reel they measure fields up to 0.1 nT.

#### 3.1.3. Applications

This kind of sensor is already known for 60 years [20]. The first magnetometers were developed during the thirties to be used in magnetic studies of the atmosphere or during the Second World War to detect submarines. Afterwards they have been used to find minerals or for magnetic measurements in the outer space. They have also been adapted for detection and surveillance devices and for the non-destructive characterization of minerals.

Unlike magnetometers used for detection or geometrical earth measurements, space magnetometers must not be change detectors but absolute measurement instruments with a wide dynamic range of operation. They show important restrictions due to their weight and consumption and must guarantee a correct operation even under big temperature fluctuations. The need for absolute measures of the planetary fields requires a high zero level stability (which implies minimum drifts), low noise level within the measuring instrument and a good thermal stability [23,24,25].

Other types of magnetometers are used for surveillance (weapon detectors) and process control (motor speed, passage detection *etc.*). For these applications, compact, cheap and low consumption devices are required, even if their sensitivity and stability is worse, because they can be frequently adjusted without great efforts.

(a) Archaeomagnetism

Archeology also takes advantages of magnetometric sensors. Some ruins have natural magnetic marks that can be detected by means of fluxgate sensors even if they are buried. The earth’s magnetic field is always present on the earth’s surface. This (vector) field is of 50.000 nT. Ruins research is done by the observation of field distortions. For example, the remainders of the end of the Bronze Age in North America and Rome distort the field between ±10 and 100 nT; earlier remainders (Neolithic, beginnings of the Bronze Age) can distort it between ±0.05 and 10 nT. Depending on the desired tracking, a minimum sampling resolution is necessary [26].

(b) Magnetic Mapping

Although archaeomagnetism can also be considered as a magnetic mapping method, this section concerns the creation of maps for space missions. Space technology has used magnetometric sensors [27] almost from the beginning. They have been used in Apolo missions for the drawing of moon maps, for creating altimeters and navigation and positioning systems, *etc.* This section relates to a specific mission: the Space Technology 5 (ST5) [28].

The ST5 is part of a set of missions, named by the NASA “New Millennium Programme”, with the specific goal of establishing the Earth’s magnetosphere, making use of a network of nanosatellite sensors planned to be launched in 2003 (Table 1).

**Table 1:** Relevant data for the magnetometric sensors used in ST5 missions.

Parameters	Values
Total mass:	361 g + 250 g (frame)
Sensor mass:	75 g
Sensor volume:	4 × 4 × 6 cm
Energy consumption, Electronic:	500 mW
Energy consumption, Sensor:	50 mW
Range selection:	1000, 64.000 nT
Data rate:	16 vectors/s
Data resolution:	18 bits (1:range, 1:sign, 16:value)

Maybe the magnetometric system of these nanosatellites is the most interesting part, since it constitutes the evolution of five different kinds of fluxgate magnetometers. These magnetometers are: ISEE, Galileo, Polar, FAST y FedSat. The following Table 2 includes a comparison of their properties.

**Table 2:** Comparison between fluxgate magnetometers used in different missions.

Mission Name	Range (nT)	Cadence (nT)	Mass (g)	Area (in^2^)
**ISEE**	8000, 556	4, 16	500	100
**Galileo**	16.000, 512, 32	0.05, 3, 32	500	100
**Polar**	47.000, 5700, 700	8.3, 100	400	70
**FAST**	64.000	Up to 512	350	60
**FedSat**	60.000	10,100	200	35

Finally, a FedSat magnetometer control unit weights around 200 g and supports an operating range of 60.000 nT, with sample frequencies of 10 or 100 Hz.

(c) Security Systems

Undoubtedly, magnetometric sensors most in use are those destined to security systems. Basically, there are two different kinds of security systems: weapon detection and theft detection. One of the most sophisticated weapon detection systems with fluxgate sensors was developed by the Idaho National Engineering and Environmental Laboratory (INEEL) commissioned by the U.S. Army and commercialized by Milestone Technologies (Idaho Falls, ID, USA) under the brand SecureScan2000.

The SecureScan2000 measures the magnetic field variations of the earth when passing by and detects weapons with great accuracy. To avoid detection of other objects (keys, telephones, *etc.*) the system starts from the assumption that all weapons carry magnetic materials (iron or similar). It combines the result of the magnetic scan with a real image and shows it on a screen, indicating the exact location of the weapons. An alternative weapon detector is the hand detector. To avoid theft in department stores, clothing shops, libraries and similar places, panels can be equipped with magnetometers [29] that are able to detect magnetic tracks.

#### 3.1.4. Planar Micro-Fluxgate

When reducing the dimensions of a fluxgate, the device loses sensitivity as a consequence of noise increase. However, when reducing proportionally the microfluxgates’s area, its sensitivity does not diminish. The main problem of miniaturizing fluxgates stems from the thickness and the volume of their coils.

(a) CMOS Technology

For these microfluxgates, planar technologies are applied (specifically CMOS) to implement the necessary circuitries and coils. The chips currently available on the market are equipped with two integrated orthogonal microfluxgate sensors with their corresponding A/D conversion circuitry. The application of coils in microfluxgates is perhaps the most original thing about them.

They are created in the shape of various metal overlapping layers separated from each other by dielectric material (silicon oxide) [30].

They measure magnetic fields in a similar way as other fluxgate magnetometers: saturating ferromagnetic cores (actually, nickel-iron-molybdenum cores) and measuring the lag produced by external fields. These kind of devices have the following advantages compared with conventional fluxgates:

- They need little space.
- They provide linear responses for overhead magnetic inductions (there are certainly “conventional” fluxgates that offer a better response, but not in such a reduced area of 50 μT).
- They measure the chip plan (360°) without ambiguity.
- Their production is easy and very cheap using planar technology.
- They have the whole control circuitry integrated.

However, in spite of these advantages, one must keep in mind that these sensors are specifically calibrated to measure the earth field. At least for the moment, there are no commercial microfluxgate sensors able to function in other magnetic ranges. Their maximum linear output range is of ±100 μT.

Technological development concerning microfluxgates has brought on the market different chip models. For example, the chip developed by Chiesi *et al.* [31]. This microfluxgate carries a circuitry set up with the standard CMOS process and a ferromagnetic core produced thereafter. 

As for the Chiesi chip, the developed micro-sensor consists essentially of a parallel fluxgate magnetometer of two axes. The uniqueness of the setting lies in the geometric layout of its elements, which allows the use of one single coil to saturate the two cores, optimizing the length of the latter. The trick consists in locating the two cores crosswise and perpendicularly to each other, above the square coil. This way the secondary coils are located at the ends of both cores. For a given exciting flow, with this arrangement the core magnetization increases up to a 30% compared with the classic setting.

The magnetic cross shaped core is realised by means of photolithography and chemical attack of amorphous tapes and subsequently attached to the previously produced chip with CMOS technology [32,33]. Both secondary coils, located under the core ends, are series connected along the measuring axes with the objective of obtaining a differential configuration. This cancels the contribution of the exciting field to the total induced signal. 

The device is powered by a 5 V supply with a frequency of 125 kHz. The exciting flow is of 17 mA_peak_, giving a consumption of 12.5 mW. The output signal shows a linear response in a range between –100 and 100 μT. The resulting sensitivity of the sensor equals 3700 V/T with an error of less than 0.4% and with an accuracy level of 1.5° in the obtained angle for a horizontal field of 9 μT 1.5°.

(b) PCB Technology

Printed Circuit Board (PCB) technology also has been widely used in the development of planar fluxgates [34,35]. Kejík *et al.* [36] have recently developed a 2D planar fluxgate composed of two orthogonal plan coils and an amorphous ferromagnetic core in the shape of a PCB ring. Each of the coils has a density of 50 spires/cm and the core consists of a Vitrovac 6025 tape realised by means of photolithography. The detection principle is based on alternating measurements in two directions. In each case, the exciting coil is orthogonal to the measuring axis. As a result, the output voltage of the secondary coil only contains the external field signal. The authors report that the sensor is linear in a range of ±60 μT, showing a sensitivity of 55 V/mT. The application of the sensor is determined by its capacity to measure the components of the Earth’s magnetic field, as it were, like an electronic compass. The accuracy level in the obtained angle for a horizontal field of 30 μT is of 1°.

(c) Electrodeposition Technology

Another technology combines PCB technology with the electrodeposition of Co-P alloys [37]. This system has, among others, the great advantage of avoiding the use of glue to adhere the core to the device. For example, the device developed by Dezuari *et al.* [38] is based upon various one and double faced plates in which the tracks are obtained by means of photolithography. This method allows to produce fluxgates with a maximum sensitivity of 160 V/T with a flow between 300 mA and 10 kHz.

(d) Hybrid Magnetometers—Magnetoelectric Sensors

In the last decade a set of hybrid magnetic sensors has been developed of piezoelectric and ferromagnetic material as an alternative to traditional sensors [39], they possess competitive characteristics for the present-day market.

Piezoelectric materials respond to electric excitation with relative size changes and ferromagnetic materials respond likewise in the presence of a magnetic field. The range of these relative changes depends on the material’s intrinsic characteristics. Moreover, the size of a ferromagnetic substance only changes when magnetization occurs by means of rotation; when it occurs through wall movements of 180° there is no global change of size since the latter separate already saturated domains. In order to produce in a magnetostrictive ferromagnetic substance a significant change of size, it is convenient to put it previously under a thermal treatment that positions the easy magnetization direction perpendicularly to the longitudinal axis (axis in which the sensor is magnetized).

When a piezoelectric substance and a highly magnetostrictive ferromagnetic substance are united mechanically, the size changes produced in the piezoelectric material resulting from an electric field are transmitted to the ferromagnetic substance, affecting its inner structure and hence, its magnetic behaviour. Logically the opposite effect is also possible. This way magnetic type changes take place with electrical excitation or *vice versa*. These cross effects can be used to detect external magnetic or electric fields and to measure some qualities of the hybrid's constituting materials.

In order to detect weak magnetic fields by means of a piezoelectric ferromagnetic hybrid, various settings are possible. The most common configuration is the following: the piezoelectric material is a support with quite a bigger size than the ferromagnetic material, excited by an electric field outside its longitudinal resonance frequency (at this frequency the relative size changes on the longitudinal axis are considerably greater than at any other frequency). Ferromagnetics have a high level of magnetostriction, so size changes affect them a lot. They are also magnetically soft, this means that they are easily magnetized in presence of weak fields. Both materials are united by a viscous fluid that allows vibration transmission of the piezoelectric material, avoiding the mechanic tensions, typical of rigid glue, in both of them, but especially in the ferromagnetic material which is the thinnest one.

So, each time the piezoelectric base stretches or contracts, an anisotropy axis is induced in the ferromagnetic material, this is, a privileged axis where magnetization tends to accommodate to minimize energy. For example, if the ferromagnetic material has a positive magnetostriction, each time the piezoelectric material stretches, the magnetization tends to follow a longitudinal direction and each time it contracts, the magnetization will occur in any direction perpendicular to the longitudinal axis. In the presence of a magnetic field, the ferromagnetic material will magnetize more easily when the piezoelectric material stretches and it will magnetize less easily when the piezoelectric material contracts. These size changes happen at the resonant frequency (tens of kHz), accordingly, if a secondary coil is rolled around the core, the signal induced is a sine wave with an amplitude that depends on the piezoelectric vibration range, on the external magnetic field range and on various other parameters of both materials. 

Using these data, a magnetic field sensor with excellent features can be made. With this kind of sensor, magnetic fields as small as 100 pT can be measured, thanks to its great sensitivity and low noise level. The frequency range in which the hybrid sensor operates extends to frequencies close to the resonance of piezoelectric materials. It is also highly stable in time, being its stability limited by thermal effects that damage the interface’s sticky fluid. Furthermore, it is small of size, easy to produce, low cost and a low consumption (of the order of mW).

More recently, some authors have developed different magnetoelectric (ME) sensors [40,41,42]. In [40] a theory for non-linear ME effects in planar ferromagnetic-piezoelectric composite structures is described. It was shown that non linearity of magnetostriction results in the generation of dc voltage across the piezoelectric, doubling of ac magnetic field frequency, and generation of ac voltages with sum and difference frequencies.

In [43] we can see a ME sensor built by encapsulation in vacuum. This micro-sensor has a sensitivity of 3800 V/T and a resolution of 30 pT, which is comparable to larger sensors ME [44] and magnetic biosensors.

### 3.2. Bistable Sensors

#### 3.2.1. Theory

The phenomenon of magnetic bistability in amorphous substances was observed firstly in iron-rich and highly magnetostrictive threads. The magnetic and mechanical characteristics of these threads and their possible technological application have been examined in depth. Several authors have studied these materials to establish the dependency of the Young modulus on the applied magnetic field, the longitudinal and transversal magneto-resistance, the magnetostriction, *etc.* [45,46,47,48].

However, the basic mechanisms responsible of magnetic bistability were not determined immediately. Therefore, the bibliography includes many papers of related to various characteristics of these threads, amongst them the Barkhausen and Matteucci effect, the influence of mechanic tensions on magnetization processes and observations of magnetic domains with the Bitter technique.

The reason why the threads show a bistable behavior is that, due to the manufacturing process, two areas appear: the inside area that is tense and the magnetization of which is aligned with the thread’s axis; and the outside area that is compressed with a magnetization perpendicular to the axis. As a result of this setting, a strong magnetoelastic coupling, responsible of the bistable behaviour, occurs. Initially, the bistable jumps were attributed to the spread, alongside the thread, of a conic magnetic wall that was thought be nucleated at the thread ends like a closing domain to decrease its magnetostatic energy. Spread measurements of these walls were realised. Today, taking into account the braking measurements through induced flows of the propagation wall in the thread, it seems more likely that the wall spread has a plane and not a conic propagation. 

Since the bistable behavior is a consequence of a magnetoelastic coupling, the parameters of the loop described by the samples under the action of an alternating magnetic field will be strongly influenced by tensions applied from the outside. The thermal treatment of these threads subjected to tension will influence their magnetic features. Likewise, it is possible to obtain bistability on non-magnetostrictive threads by merely applying tensions and torsions to reinforce the magnetostrictive coupling between the inner and outside areas of the thread.

Also the influence of an axial electric flow on the magnetization processes of bistable threads has been studied by many different authors, obtaining a shift of the bistable jumps which is a function of flow intensity [49]. These bistable jumps can be extinguished by applying simultaneously axial tensions, torsions and electric currents.

#### 3.2.2. Obtaining and Features

**Figure 11:**
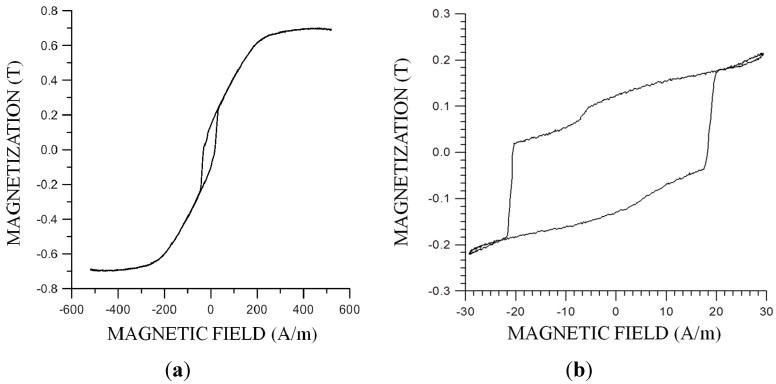
Bistable samples: (**a**) hysteresis loop; (**b**) Minor loop.

We have observed in [49] that amorphous bands of low magnetostriction, Metglass 2705 M (Co_70_Fe_5_Ni_2_Mo_5_B_3_Si_15_, λ_S_ ≈ −0.3 × 10^−6^, 8 cm length, 1.2 mm width and 20 μm thickness) previously annealed during 15 min, with a continuous current of 750 mA (J = 3.125 × 10^7^ A/m^2^ heated up to 250 °C) show hysteresis loops like in Figure 11a with two great irreversible jumps in the central area, when subjected to alternating longitudinal magnetic fields, evolving the magnetization of the rest of the loop as if there were only coherent rotations and no applicable hysteresis.

These two big irreversible jumps remain unchanged when the magnetic field range decreases, as is shown in Figure 11b; but if the field under the sample’s coercive field decreases, loops are not smaller. The magnetization stays above or under the minor loop. Concluding, the samples present magnetic bistability, having two possible magnetization states when no magnetic field is applied.

#### 3.2.3. Applications

(a) Codification and Magnetic Cards

The magnetic behaviour of highly magnetostrictive threads can be recognized by the abrupt appearance of a Barkhausen jump during the magnetization process of the material. Using a conventional induction system, the appearance of the Barkhausen jump as a consequence of the fast magnetization inversion in the inside core of the thread, produces a well-defined pulse on the voltage in the secondary ends. Through combination of threads with different critic fields and assigning a bit to each pulse, a magnetic codification system can be organized.

One of the difficulties of this system is that the threads interact mutually; this means that each thread creates in its environment a dispersion field which modifies the value of the pulse corresponding to the thread’s magnetization inversion, hindering the magnetic reading. Vázquez [50] studied the movement of the voltage pulses by introducing in the secondary end two highly magnetostrictive, Fe-rich threads of 10 cm long. The measurement was realised with the help of a conventional induction system of 18.7 Hz. If the two threads did not interact, the two pulses would appear simultaneously, which was not the case during the experiment.

However, if codification is done taking into account only the height or maximum voltage of the voltage pulses instead of the position of each of them, all the previously described technical difficulties disappear. In this case, it would only be necessary to detect the number of pulses, as well as the maximum value of each of them. Accordingly, by using three threads, 2^3^ = 8 possibilities are obtained. Thus, by increasing the number of threads, the codification possibilities multiply considerably.

Another problem of bistable Fe-rich threads is that a considerable remanence increase generally involves a deterioration of the bistable component (internal tensions’ relax). However, non-retostrictive Co-rich threads annealed under low tensions allow, in the first place, to reproduce the bistable behaviour observed in high magnetostriction threads. For the specific application under consideration, what matters is that the tension variation can produce important variations of both the magnetization fraction that participates in the jump and the critical field itself. An increase of the annealed tension augments the remanence and decreases proportionally the critical field. On the other hand, since the saturation magnetization is approximately the half as in the Fe-rich thread, the effects associated with the interaction of the threads along the dispersion field will be inferior. There are various possibilities, for example, three threads of a non magnetostrictive Co-rich alloy subjected during 5 min to a current density of 28.5 A/mm^2^ and to different annealing tensions; being V_1_, V_2_ and V_3_ the voltages corresponding to the annealed threads with 240, 160 y 80 Mpa respectively. 

What matters about the obtained results is that regardless of the interaction effect between the samples, the maximum voltages of the three peaks can be identified easily, for all cases, within the following intervals:

43 < V_1_ (mV)  24 < V_2_ (mV) < 34  10 < V_3_ (mV) < 15

Accordingly, these results evidence that non magnetostrictive threads subjected to tensions can be used for the identification of magnetic cards. The latter consist of the following: bistable threads are stuck to a card that is to be introduced in a device which is basically manufactured with a rectangular (primary) coil that creates an alternating field alongside the thread’s axis and a secondary detector that, when introducing the card, recovers the signal from the samples’ central area. The whole system is perfectly screened in order to eliminate the effect of external magnetic fields. The primary coil can be powered by a 50 Hz current of a transformer simply connected to the mains supply. Fourier spectrum analysis of the signal obtained in the secondary coil contains a response of rich and varied harmonics.

(b) Signature Validation Pen

In [50] a magnetoelastic sensor for verifying and validating signatures is presented; its magnetic behaviour depends on the intensity and order of the applied mechanical tensions during the signing process.

It is common knowledge that the signature of a person can be represented by a series of tensions. The sequence and strength of these tensions are typical of each person’s signature. Consequently, the temporary tension changes while signing can be used to identify a signature. This effect has been used to create a signature validation pen, consisting of a ferromagnetic bistable thread with positive magnetostriction, a miniaturized secondary coil and a simple mechanism; the secondary coil transfers the applied tensions to the thread. The main characteristics of the temporary tension changes corresponding to a signature are signature time, sign and peak sequence detected.

(c) Position Sensor

The position sensor is based on the spreading mechanism responsible for the appearance of bistability in the magnetic behaviour of magnetostrictive amorphous threads [51]. Assuming that the spreading velocity remains practically constant alongside the thread, by fixing the location of one of the secondary detectors, the position of the other secondary coil can be determined. In this case, the sensor’s sensitivity is limited by the precision of time determination. A significant decrease of the spreading speed of the wall will produce for the same distance, an increase of time and thus an increased accuracy in distance determination (sensitivity = μm).

### 3.3. Magnetostrictive Sensor

#### 3.3.1. Theory

A stable magnetization direction M in a ferromagnetic sample is determined by the configuration that most minimizes the total free energy of the system. Considering that the saturation magnetization M_S_ of a thin film forms an angle θ with the longitudinal axis of the sample, that a H_R_ field forming an angle α with the axis is applied and that the anisotropy field forms an angle γ with the said axis, the system’s magnetic energy will be defined as follows [52]:

(1) Zeeman Magnetic Energy

When applying an external magnetic field, magnetization will tend to parallel that field in order to decrease the system’s energy:

$$E_{H}\;=\;-\;M_{S}\;H_{R}\;\cos\;\left(\alpha \;-\theta \right)$$

(2) Anisotropy Energy

Anisotropy energy concerns the existence of different preferential directions, so-called easy axis, to magnetize the material. These easy axes are determined by the rate of symmetry of the crystalline pattern of the (spin-orbit coupling):

(4) $$E_{A}\;=\;+\;\frac{1}{2}\;M_{S}\;H_{K}\;\sin^{2}\left(\gamma \;-\theta \right)$$

(3) Magnetostatic Energy

It represents the magnetization energy in the demagnetization field created by magnetization itself as a consequence of the existence of pole distribution in the sample:

(5) $$E_{D}\;=\;+\;\frac{1}{2}\;{M^{S}}_{2}\;N_{D}\;\sin^{2}\theta$$

being N_D_ the demagnetization field opposite to M_S_ direction:

(6) $$E_{A}\;=\;+\;\frac{1}{2}\;M_{S}\;H_{K}\;\sin^{2}\left(\gamma \;-\theta \right)$$

adding up the three contributions to the system’s energy and differentiating with respect to θ, we can find the condition that minimizes that energy:

(7) $$H_{R}\;\sin\left(\alpha -\;\theta \right)-\;\frac{1}{2}\;H_{K}\;\sin2\left(\gamma -\;\theta \right)-\;\frac{1}{2}\;N_{D}M_{S}\;\sin2\theta =0$$

**Figure 12:**
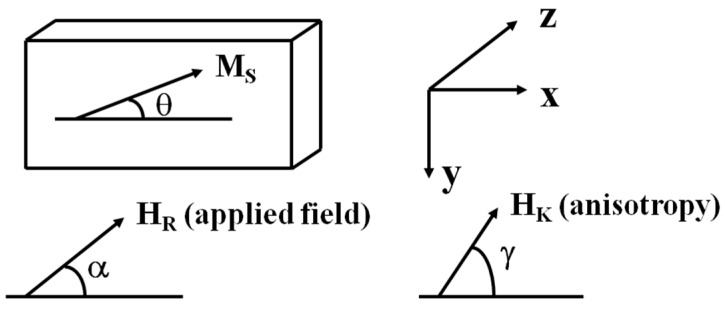
Magnetization, applied magnetic field and anisotropy direction.

This equation determines the relation between the applied field H_R_ and the θ angle between the magnetization vector and the longitudinal film axis, which is also used as the current direction (Figure 12). This θ angle has the following relation with the material’s resistivity:

(8) $$\rho =\;\rho_{0}\;+\;∆\rho \;cos^{2}\theta$$

being Δρ the maximum value of the resistivity change to the magnetic field (saturated magnetoresistance):

$$H_{R}\;\sin\;\left(\alpha \;-\;\theta \right)\;-\;\frac{1}{2}\;H_{K}\sin\;2\left(\gamma \;-\;\theta \right)\;-\;\frac{1}{2}\;N_{D}M_{S}\sin\;2\theta \;=\;0$$

Combining Equations (7) and (8), θ can be eliminated, obtaining so the relation between the relative change of magnetoresistance and the magnitude and direction of the applied field, assuming that the other constants are known. This is the operating basis of magnetostrictive sensors.

#### 3.3.2. Applications

(a) Magnetic Field Sensors

**Figure 13:**
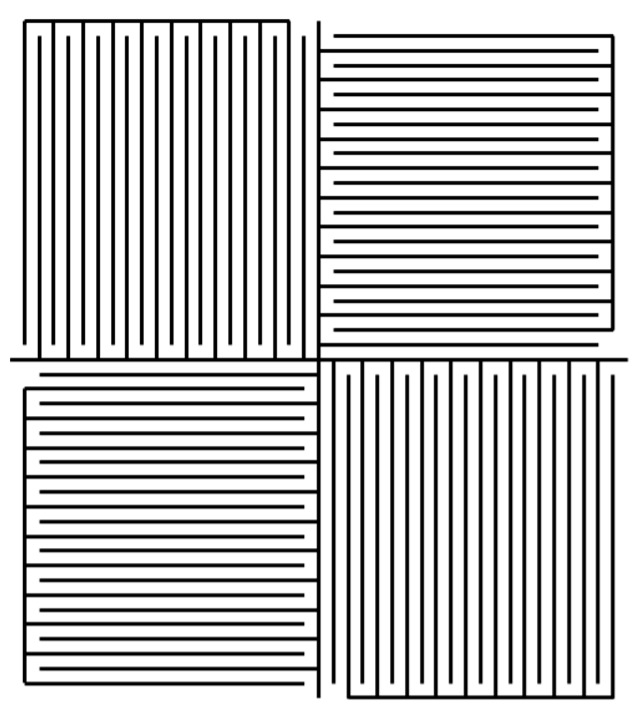
Resistance bridge with conductive bands forming a 90° angle between them.

It is of common knowledge that resistivity of magnetic materials depends on the angle between current direction and magnetization direction, this effect being of the order of 2% in NiFe alloys, which allows to measure magnetic fields with a high resolution [53]. The magnetostrictive effects are used in magnetometers, reading heads, position sensors, angle sensors, *etc.* Figure 13 illustrates one out of many possible device configurations. A thin film is properly attacked to form a resistance bridge, the conductive bands forming a 45° angle with the field to be measured (north-south direction) and with the sample’s easy axis (east and west direction). When changing its magnetization direction, the magnetic field unbalances the bridge, compensating the thermal effects.

When magnetization parallels the current, the potential difference in the transversal contacts is 0: when the magnetization direction changes by the action of a magnetic field, the material's magnetoresistance causes the appearance of tensions in those contacts. This phenomenon is known as the Hall planar effect and reaches its maximum value when magnetization forms a 45° angle with the current. The sensitivity of this device depends on the sample’s anisotropy and on the magnetoresistive constant, therefore amorphous bands of high magnetostriction, low anisotropy and low anisotropy dispersion are used for these sensors, comparable to the use of extensometric bands.

This sensor has been used to measure peak magnetic fields, with a sensitivity of 10 nT/µV, just like all other magnetometers coupled to magnets for measuring angles, distances, *etc.* The sensitivity of magnetostrictive sensors has increased thanks to the use of technologies which have managed to reduce the sensor’s thickness. So, 150 Å thin films can produce a uniform magnetoresistance with the applied field, achieving signal/noise rate of 97 dB. The thickness is very important because very thin films have Néel walls instead of Bloch walls. Because of their greater thickness Néel walls pass imperfections with only small energy changes, producing a considerably lower noise level than Boch walls. In order to improve the performance of these sensors the microstructure of their walls has been regularized by including a sublayer of various materials such as platinum or silver (improving the signal/noise rate), sophisticated electronic circuits have been implemented to improve the signal quality and hysteresis has been ”eliminated” applying high frequency field.

(b) Sensors of Anisotropic Magnetoresistance 

One of this assortment of sensors is the anisotropy magneto resistance sensor, able to reach relative resistivity variation of 3.93% in Permalloy films of 50 nm thickness. Bridges manufactured with this material have a 0.5 V/nT sensitivity in a range of Mhz [54].

(c) Giant Magnetoresistance Sensors

Finally, planar technology has been applied to develop giant magnetoresistance sensors. Some bridges, for instance, show sensors with a resistance bridge of various antiferromagnetic FeNi/Ag layers which provides a relative resistance variation of 10% and a bridge sensitivity of 0.6 mV/V/Oe with a linearity of less than 1%.

Schotter *et al.* [55] manufactured a biochip based on huge magnetoresistance sensors. With the aim of analyzing the molecular composition of a given sample, magnetic marks are specifically confined in molecules, the magnetic marks field being detected by a magnetostrictive sensor. The magnetic marks are superparamagnetic microspheres which are available on the market with an average diameter of 0.86 μm.

The giant magnetoresistance sensor consists of 30 sensor elements, each of them manufactured with NiFeCu multilayers in spiral shape with a maximum diameter of 70 μm. Therefore each device allows the simultaneous analysis of 15 DNA tests.

Being superparamagnetic, the magnetic microspheres must be magnetized to produce a signal on the sensor. As the sensor only responds to the fields of the film plane, that response will be due exclusively to the components of the parallel field along the plane induced by the magnetized microspheres. So, each sensor element will produce information of the size of the surface covered by the magnetic marks, which makes it possible to deduce the concentration of molecules correspondent to the DNA test, enabling the detection of as much as 5% of the cover surface corresponding to a total of 200 magnetic marks.

## 4. Changes on the Inductance

### 4.1. Linear Variable Differential Transformer

#### 4.1.1. Theory

This type of sensor is based upon the mutual induction coefficient variation between two coupled circuits with a magnetic core, as a consequence of a position change of this core [56].

**Figure 14:**
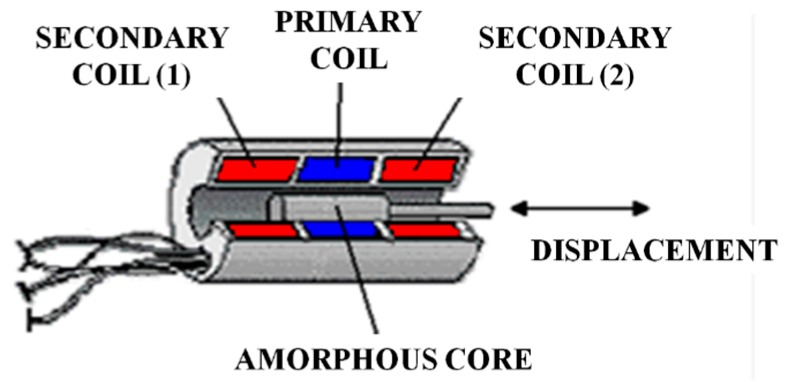
LVDT experimental set-up.

The linear variable differential transformer is commonly referred to as LVDT. This sensor consists of a primary winding alongside the core centre and of two secondary coils placed symmetrically in relation to the centre (Figure 14). The three windings are covered with an impermeable substance to enable them to operate in highly humid environments. The core is an iron and nickel alloy, longitudinally laminated to reduce the Foucault currents. The stem that drags it should not be magnetic, and is at the other end attached to the piece of which the movements are to be measured. The whole set can be magnetically screened to make in it immune to external fields.

**Figure 15:**
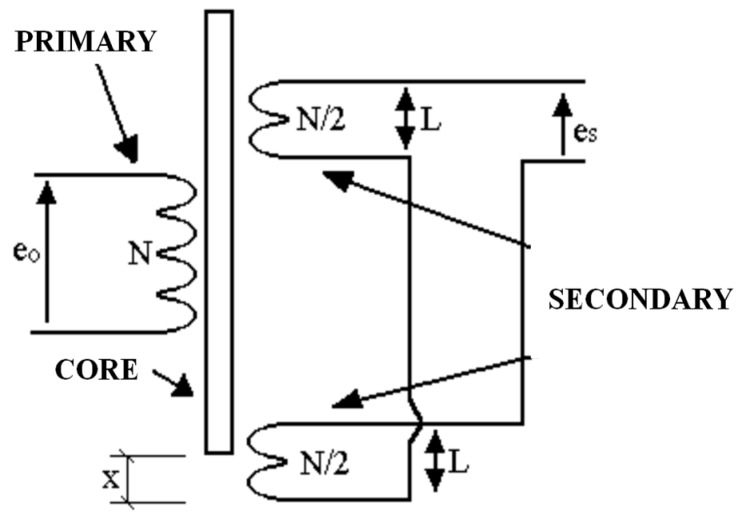
Electronic diagram.

When an alternating current is applied to the principal winding, a current is induced into the two secondary coils. Since the secondary coils are connected in series-opposition, when the ferromagnetic core is in the centre, the tensions induced in each secondary coil will be equal and the output sensor will give a zero value. When the core moves away from the centre, one of the two tensions increases and the other one reduces with in same magnitude (Figure 15), generating a proportional linear output to the magnetic core position. The relation between input and output tension can be determined by means of basic transformer equations, as they basically function in the same way.

(9) $$\frac{e_{s}}{e_{0}}=\;\frac{\frac{N}{2}-\;\frac{N}{2}\;\left(\frac{L-x}{L}\right)}{N}=\;\frac{x}{2L}$$

where *e_s_* is the output voltage of the secondary coils and e_0_ is the primary coil input voltage.

The main problem with this traducer configuration is that it becomes impossible to detect the sense of movement within it. In order to solve this problem, the transducer is provided with a suitable signal fitting. For that purpose, a phase detection is carried out.

In principle, the e.m.f. of the secondary coils increases with the frequency. However, according to the “skin” effect, the real response of the device gives a maximum value, because the penetration depth of the magnetic field into core decreases with the frequency. That is why usually hollow cylindrical cores are used, since with the normal operating frequencies of these devices (from 1 to 5 KHz), the magnetic field hardly penetrates in ferromagnetic conductor materials.

This device can reach a 0.25% to 0.05% linearity in a millimetric range, depending on the core length and the excitation frequency. With a proper design it is possible to measure 10 mm with micron resolution.

#### 4.1.2. Applications

LVDT are mainly used as position sensors and, when modifying their reels and core shape, as angle sensors. LVDTs with E shape [57] are used as remote position sensors, able to operate even with a wall in between the reel system and the magnetic core.

Indirectly they are used as deformation sensors, as for example on beams deformation measures or in some deformation tension measuring devices [58]. The same way, they are used to measure deformations on pressure sensors diagrams or, coupled to a resort, like strength sensors.

**Figure 16:**
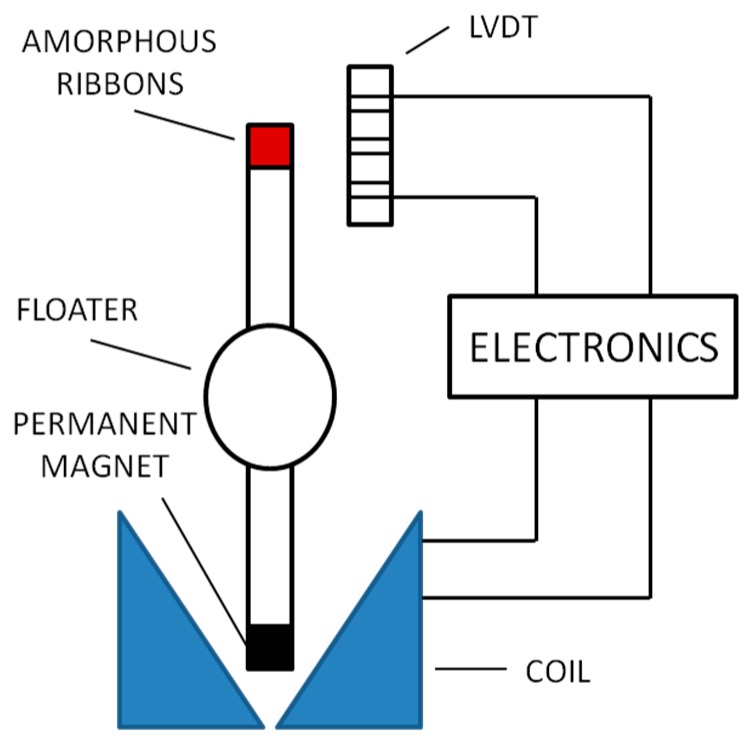
Density sensor [60].

As an example of the versatility of this kind of sensor, Figure 16 shows a density sensor that uses a LVDT and a small magnetic actuator [59]. The LVDT acts as a zero device that feeds back to the magnetic actuator so that the small hollow sphere can maintain a static levitation in the fluid.

Another LVDT version consists of a ferromagnetic core, linked with the movable part that moves around a solenoid, changing its self-induction coefficient with respect to the core position. Its value must by read by means of alternating current bridges or in a differential way [60,61].

### 4.2. Sensors Based on Giant Magnetoimpedance

#### 4.2.1. Theory

The magnetoimpedance is defined as the change in impedance of a ferromagnetic conductor, to which is applied an AC current of low intensity and constant frequency, in order to induce an alternating magnetic field in the presence of an external magnetic field. Changes in impedance are caused by the variation of the material magnetization with constant magnetic field and the alternating magnetic field induced by alternating current. Accordingly, these changes in impedance reflect changes in the magnetic structure of the material. So the increase in impedance is a function of the permeability of the material in the direction of the applied field [61].

Early work correctly describing the effect magnetoimpedance was published in 1930 by Harrison, based on experiments performed in Permalloy wire. However, it was in the year 1990 when this topic attracted interest due to the discovery of amorphous and nanocrystalline magnetic materials, resulting in a significant number of publications. The first published magnetoimpedance effect work in amorphous samples was developed by Makhotkin in 1991. Subsequently, the giant magnetoimpedance was defined as the response of materials at high frequencies, so that the surface effect results in large changes in impedance of 600% in some materials [61].

For high frequencies, the effect of giant magnetoimpedance (GMI) it has been explained by classical electrodynamics, as an interaction between the alternating magnetic field and the magnetization due to the constant magnetic field. The GMI effect is mainly caused by changes in the resistive component of the impedance, as is a skin effect. This causes current is limited to the sample surface, reducing the effect of the cross section of the material, producing an increase in the resistive component of the impedance. Therefore, at high frequencies the skin effect is determining the behavior of the magnetoimpedance.

#### 4.2.2. Applications

(a) Magnetic Field Sensor

In work [62] it can see a magnetic sensor formed by two parallel microwires. These microwires are connected in series and allows to measure continuous and alternating fields with a sensitivity of 10 mV/(A/m). This sensor was further improved by increasing the range for continuous fields up to 1 kA/m with a compensation coil. Another way we can find in [63] a new amplitude detector. It is formed by a device (Direct Digital Synthesizer) connected to a GMI sensor, that produce a voltage which is applied to detector. The output of this detector is proportional to the magnetic field measured and the sensitivity is around 0.0164 V/A/m.

(b) Biosensors

The high sensitivity of GMI sensors positioned them in an ideal position for use in the development of magnetic biosensors. A magnetic biosensor detects magnetic field variations caused by magnetic microparticles used as biomarkers [64]. Compared to other detection techniques such as fluorescent markers or electrochemical measurements, the use of magnetic biomarkers has many advantages.

The device designed by Wang *et al.* [65] has been tested in tumor tissues and identifies gastric cancer cells. The biosensor comprises GMI sensors and magnetic nanoparticles. The magnetic nanoparticles are attached to cancer cells so that when an external magnetic field is applied nanoparticles align with the field and cause a change in the magnetoimpedance sensor. More recently an integrated giant magnetoimpedance biosensor for detection of biomarker has been developed [66].

## 5. Conclusions

Since the introduction in 1960 of the first metallic amorphous material, study and development of such materials has been an area of great interest because of their basic characteristics, such as lack of translational periodicity and non-directional nature of the link. This was a great break from previously known materials. Next to this aspect, of fundamental interest, amorphous materials have a great potential from the point of view of its technological applications; as is well known some of these materials exhibit outstanding properties mechanical, chemical, *etc.*

One of the fundamental characteristics of amorphous, from the point of view of their possible technological applications is the nature of soft materials. They have not magnetocrystalline anisotropy, so the main source of anisotropy is the tensions introduced by the manufacturing process, being much of these stresses removable by heat treatments. As a consequence have high sensitivities (about 10^5^) and low coercive fields (up to mils Oe); also with a resistivity, approximately one order of magnitude higher than the corresponding crystalline state, it is produce that eddy current losses decreases. 

Moreover, the large number of existing compositions and the possibility to vary their properties by different treatments, within certain limits, the most suitable material for a particular application. In fact, amorphous ferromagnetic materials have displaced virtually as other more conventional crystalline ferromagnetic semiconductor, ferroelectric, *etc.*, in the development of new sensors very robust needed to automobiles, robotics, industrial measures, *etc.*, where the requirements are extremely severe. 
